# The Nogo Receptor Ligand LGI1 Regulates Synapse Number and Synaptic Activity in Hippocampal and Cortical Neurons

**DOI:** 10.1523/ENEURO.0185-18.2018

**Published:** 2018-09-07

**Authors:** Rhalena A. Thomas, Julien Gibon, Carol X. Q. Chen, Sabrina Chierzi, Vincent G. Soubannier, Stephanie Baulac, Philippe Séguéla, Keith Murai, Philip A. Barker

**Affiliations:** 1 Department of Neurology and Neurosurgery, Montreal Neurological Institute, McGill University, Montreal, Quebec H3A 2B4, Canada; 2 Department of Biology, University of British Columbia, Kelowna, British Columbia V1V 1V7, Canada; 3 Department of Neurology and Neurosurgery, Centre for Research in Neuroscience, The Research Institute of the McGill University Health Centre, Montreal General Hospital, Montreal, Quebec, H3H2R9, Canada; 4 Université Pierre-et-Marie-Curie Université Paris 06, Sorbonne Universités, Paris, 75005, France; Institut National de la Santé et de la Recherche Médicale, Unité 1127, Paris, 75006, France; Centre National de la Recherche Scientifique, Unité Mixte de Recherche 7225, Paris, France; Institut du Cerveau et de la Moelle Épinière), Paris, 75006, France; Groupe Hospitalier Pitié-Salpêtrière, Paris, 75013, France

**Keywords:** epilepsy, leucine-rich glioma-inactivated gene 1, RhoA, synaptic activity, synaptic plasticity, TROY

## Abstract

Leucine-rich glioma-inactivated protein 1 (LGI1) is a secreted neuronal protein and a Nogo receptor 1 (NgR1) ligand. Mutations in LGI1 in humans causes autosomal dominant lateral temporal lobe epilepsy and homozygous deletion of LGI1 in mice results in severe epileptic seizures that cause early postnatal death. NgR1 plays an important role in the development of CNS synapses and circuitry by limiting plasticity in the adult cortex via the activation of RhoA. These relationships and functions prompted us to examine the effect of LGI1 on synapse formation *in vitro* and *in vivo*. We report that application of LGI1 increases synaptic density in neuronal culture and that LGI1 null hippocampus has fewer dendritic mushroom spines than in wild-type (WT) littermates. Further, our electrophysiological investigations demonstrate that LGI1 null hippocampal neurons possess fewer and weaker synapses. RhoA activity is significantly increased in cortical cultures derived from LGI1 null mice and using a reconstituted system; we show directly that LGI1 antagonizes NgR1-tumor necrosis factor receptor orphan Y (TROY) signaling. Our data suggests that LGI1 enhances synapse formation in cortical and hippocampal neurons by reducing NgR1 signaling.

## Visual Abstract

**Figure:**
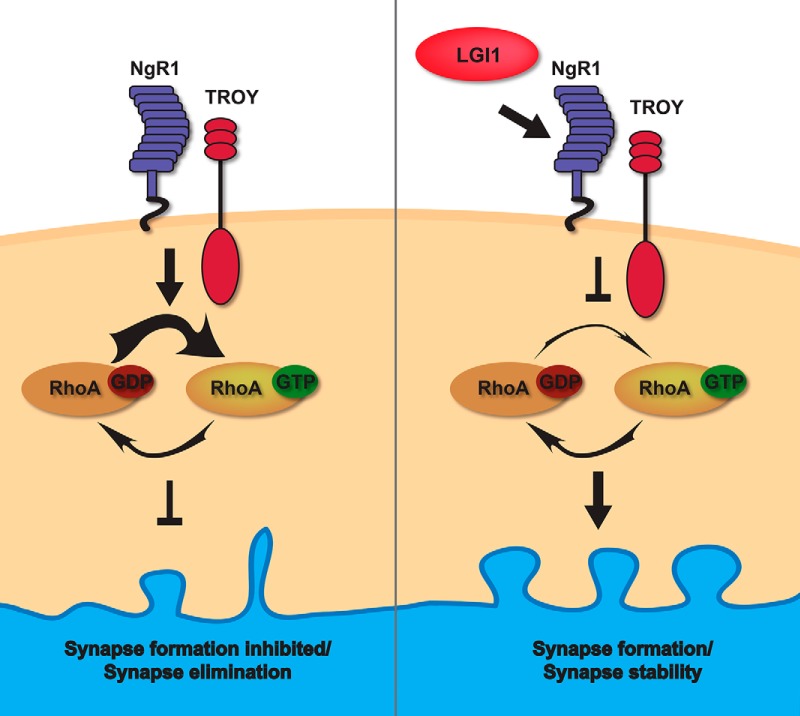

## Significance Statement

Mutations in leucine-rich glioma-inactivated protein 1 (LGI1) causes autosomal dominant lateral temporal lobe epilepsy in humans. In the present study, we used a combination of cellular imaging, electrophysiology, protein analysis, and cell biology assays to show that LGI1 promotes synapse formation and maturation. LGI1 deletion in mice results in fewer synapses and causes synaptic activity defects. Mechanistically, LGI1 regulates RhoA signaling through a receptor complex containing Nogo receptor 1 (NgR1) and tumor necrosis factor receptor orphan Y (TROY). Our experiments provide evidence that NgR1 and LGI1 balance regulates RhoA activity during synapse formation and thus impacts on synapse maturation, number, and activity.

## Introduction

During development of the CNS, numerous synapses are produced and then pruned such that only synapses forming part of coordinated and active networks are retained, while the less active counterparts are lost. The structural and signaling components that stabilize active synapses and allow them to mature have been extensively studied and key elements that regulate this cascade have emerged (Craig et al., 2006; Gerrow and El-Husseini, 2006; Kano and Hashimoto, 2009). However, the processes that drive the elimination of non-active synapses remain uncertain.

Leucine-rich glioma-inactivated protein 1 (LGI1) is a secreted protein expressed in the CNS (Senechal et al., 2005). In humans, loss of LGI1 function can have major effects on circuit activity and function, with mutations in a single LGI1 allele causing an autosomal dominant form of lateral temporal lobe epilepsy (Kalachikov et al., 2002; Morante-Redolat et al., 2002). Indeed, deletion of LGI1 in mice results in spontaneous epileptic activity from postnatal day (P)10 and death from persistent seizures by P21 (Chabrol et al., 2010; Fukata et al., 2010; Yu et al., 2010). Recent studies have shown that auto-antibodies directed against LGI1 are responsible for a subset of cases of human autoimmune encephalitis characterized by memory disturbances, confusion, and seizures in adults (Irani et al., 2010; Lai et al., 2010; Ohkawa et al., 2013).

We previously showed that LGI1 is a ligand for the Nogo receptor 1 (NgR1), acting as a competitive antagonist that blocks binding of myelin-based NgR1 ligands and thereby preventing myelin-induced growth cone collapse (Thomas et al., 2010). LGI1 is also a ligand for ADAM22, ADAM23, and ADAM11 (Fukata et al., 2006, 2010; Sagane et al., 2008). ADAM22 interacts with PSD95 and may provide a platform for LGI1 to act at the synapse (Fukata et al., 2006; Lovero et al., 2015). Interestingly, ADAM22 also binds to NgR1 (Thomas et al., 2010), raising the possibility that NgR1 and various ADAM proteins may form a complex that allows LGI1 signals to be transduced at the synapse. NgR1 was initially identified as the receptor for Nogo66, for myelin-associated glycoprotein (MAG) and olygodendrocyte myelin glycoprotein (OMGP). When occupied by any of these ligands, NgR1 forms a signaling complex with either the p75 neurotrophin receptor (p75NTR) or the tumor necrosis factor receptor orphan Y (TROY). These complexes then activate RhoA-dependent growth cone collapse and block neurite outgrowth (Fournier et al., 2001).

Intriguingly, NgR1 also plays important roles in the development of CNS synapses and circuitry. Early studies showed that NgR1^-/-^ mice have enhanced ocular dominance plasticity (McGee et al., 2005) and impairments in long-term depression (Lee et al., 2008). Mice overexpressing NgR1 have significant defects in long-term memory formation (Karlén et al., 2009). More recent work has demonstrated that NgR1-dependent RhoA activation plays a key role limiting synapse number during development (Wills et al., 2012) and that NgR1 plays a crucial role in limiting plasticity in the adult cortex in response to NogoA signaling (Akbik et al., 2013).

Both NgR1 and LGI1 are expressed in neurons throughout the brain and enriched in the hippocampus (Barrette et al., 2007; Head et al., 2007; Herranz-Pérez et al., 2010). LGI1 mRNA is found in neurons and the secreted LGI1 flag tagged protein is detected on both the cell soma and dendrite of hippocampal neurons in knock-in mice (Fukata et al., 2010). Electron microscopy analyses have revealed that LGI1 protein is present in presynaptic boutons (Boillot et al., 2016). NgR1 and its coreceptor TROY are also expressed in the dendrites of mouse hippocampal neurons (Wills et al., 2012). Here, we hypothesized that the interaction of LGI1 with NgR1 reduces RhoA signaling and thus promotes synapse formation and stabilization. In the present study, we report that exogenous application of LGI1 on primary culture of neurons increases synapse formation. In agreement with our hypothesis, we show that hippocampal neurons from NgR1^-/-^ mice form more synapses than their control neurons whereas neurons from LGI1^-/-^ form fewer synapse than their respective control in culture. In addition, an *in vivo* experiment reveals that LGI1^-/-^ mice have less mushroom-type spines than wild-type (WT) aged-matched animals. Consistent with our hypothesis, we then show that LGI1 restricts RhoA activation in primary neurons, and using a reconstituted cellular assay, we propose that LGI1 antagonizes NgR1:TROY-dependent RhoA activation.

## Materials and Methods

### Animals

All experimental procedures were approved by the McGill University Animal Care Committee and were in compliance with the guidelines of the Canadian Council on Animal Care. Animals were housed under standard conditions with a 12/12 h light/dark cycle and had free access to water and food. LGI1 mice were obtained from Dr. Stéphanie Baulac, previously reported by Chabrol et al. (2010). NgR1 mice were obtained from Dr. Sam David and Dr. Mark Tessier-Lavigne, previously described by Zheng et al. (2005). The sex of the embryos and pups used for primary cultures and hippocampal slices were not determined. Neither NgR1 or LGI1 deletion has been shown to alter sex distribution so we assume a 1:1 male:female ratio in experimental animals.

### Electrophysiology

Slices of hippocampus were prepared from C57BL6 WT (RRID:IMSR_JAX:000664) and LGI1^-/-^ littermates mice (10 d old). The brains were rapidly removed and placed in ice-cold artificial CSF (ACSF; bubbled with 95%O_2_ 5% CO_2_), which comprised: 124 mM NaCl, 2.5 mM KCl, 26 mM NaHCO_3_, 1.25 mM NaH_2_PO_4_, 2.5 mM CaCl_2_, 1.3 mM MgCl_2_, and 10 mM D-glucose. Sagittal hippocampal slices (300 µM) were prepared in ice-chilled, oxygenated ACSF using a vibratome VT1000S (Leica). Hippocampal slices were submerged in ACSF (23°C) for 2 h before transfer to the recording chamber (30°C, flow rate 1 ml/min). For whole-cell recordings, the pipette (4–10 MΩ) solution comprised: 120 mM K-gluconate, 10 mM HEPES, 0.2 mM EGTA, 20 mM KCl, 2 mM MgCl_2_, 7 mM diTrisP-creatine; 4 mM Na_2_ATP, and 0.3 mM NaGTP (pH adjusted to 7.3 with KOH). CA1 neurons were voltage-clamped at -65 mV. For miniature EPSC (mEPSC) recording, 1 µM TTX and 100 µM picrotoxin (to block GABA_A_R) was added in the ACSF. In this study, all cells had a resting membrane potential ranging from −55 to −75 mV. Cells with a resting membrane potential more positive than –55 mV were discarded. Analyses were made offline using the Mini analysis software. Neuronal excitability was assessed with a standard input/output curve obtained from an I-clamp step protocol (0–100 pA), in the presence of kinurenic acid and picrotoxin.

### Hippocampal and cortical cultures

Hippocampal neurons and cortical neurons were dissociated and cultured as described by Kaech and Banker (2006). Cortical neurons were harvested from embryonic day (E)15–E16 mice and hippocampal neurons were taken from E16–E17 mice. Astrocytes were prepared from P0–P3 WT mice and grown in astrocyte media [DMEM 2 mM L-glutamine and 100 μg/ml penicillin/streptomycin (P/S) and 15% BCS] and changed to neuronal growth media 48 h before coculturing with neurons. For quantification of synapses by immunofluorescence, hippocampal neurons were cultured on poly-L-lysine (PLL)-coated coverslips and inverted on astrocyte feeder layers for 15 and 18 d *in vitro* (DIV). Cultures were maintained in neural basal media supplemented with 2 mM L-glutamine and 100 μg/ml P/S, 1%, 2% B27. One third of the media was removed and replaced with fresh media every 3–5 d.

### Exogenous LGI1

Exogenous LGI1 was produced in 293E cells (RRID:CVCL_8869) and purified using a two-step tandem purification as previously described (Thomas et al., 2010). LGI1 protein was eluted in PBS and purity was assess by silver staining SDS page gels. The concentration of the purified protein was determined to be 16.75 µM. LGI1 was first added to complete neural basal growth media at 750 nM concentration and for control treatment an equal volume of PBS was added. Treatments were added as a one third media change for a final LGI1 concentration of 250 nM.

### Synapse quantification: immunostaining, image analysis, and quantification

Dissociated neurons grown on coverslips for 15 and 18 DIV were fixed in 4% PFA and 4% sucrose in PBS for 30 min, washed once in PBS, then briefly permeablized in methanol. After 30 min of blocking (PBS, 2.5% BSA and 2.5% goat serum), neurons were triple labeled with mouse anti-PSD95, 1:200 (Thermo Scientific, MA1-045, RRID:AB_325399), rabbit anti-synapsin1 (Syn1), 1:2000 (a gift from Peter McPherson, McGill University), and chicken anti-MAP2A, 1:2000 (EnCor CPCA-MAP2), for 2 h at room temperature. Images were obtained using a Zeiss AxioObserver Z1 inverted microscope using a 40× objective and 1388 × 1040-pixel resolution (0.161 μm/pixel). For each experiment, images were acquired as a z-stack with 19 optical sections (0.3-μm step size) followed by deconvolution (constrained iterative method). Image analysis was performed such that genotypes were blinded to the observer during analysis. Image analysis was done using NIH software ImageJ software (Fiji, RRID:SCR_002285), according to the following procedure: for every image, the threshold for each channel was defined as two standard deviations above the mean. A region of interest (ROI) corresponding to the cell body was drawn manually and excluded from further analyses in all channels. From these images, colocalization analysis was performed with the RG2B plugin and regions of double colocalization more than two pixels in size were defined as particles. To calculate synapse density, the number of particles was divided by the length of dendrites measured using a binary mask of MAP2 labeling followed by the skeletonize function (minus the cell body).

### Twiss filters

For analysis of protein levels in neurite processes, dissociated cortical neurons were seeded at high density (5 × 10^5^ cells) on PLL-coated filter inserts with astrocytes growing below the filter insert and maintained. Half the media was changed every 5 d on both the filter top and well bottoms. The protocol was adapted from Unsain et al. (2014). The filter tops containing cell bodies were harvested separately from the filter bottoms containing only axons and dendrites by the following method: filters were removed from the astrocyte-containing dish and placed into an empty dish containing PBS. The PBS was removed and the cell bodies were harvested: first by adding 100 µl of PBS to the filter top and using a cell scraper to remove cells. Cells were moved to an Eppendorf tube containing Laemmli sample buffer. Filter tops were then washed several times with PBS and wiped clean with a cotton tip to make sure all cell bodies were removed. The filter was then cut free, cut to the center, and made into a cone shape. The filter cone was then submerged in Laemmli sample buffer and boiled for 5 min. The samples were subjected to a high-speed spin, then the filters were removed and discarded. Samples were analyzed by Western blotting for actin (Fisher ICN691001, RRID:AB_2335127), Tuj1 (Covance MMS-435P, RRID:AB_2313773), LGI1 (Santa Cruz SC9583, RRID:AB_2134990), NgR1 (R&D Systems AF1440, RRID:AB_2183731), PSD95 (Thermo Scientific MA1-045, RRID:AB_325399), and synaptophysin (Syn; Sigma SVP-38, RRID:AB_2315393).

### Western blotting

Samples were boiled for 5 min, subjected to SDS-PAGE, and transferred onto nitrocellulose membranes. The membranes were incubated in TBS-Tween (TBST; 10 mM Tris (pH 8.0), 150 mM NaCl, and 2% Tween 20) supplemented with 5% (w/v) dried skim milk powder for 1 h at room temperature and then incubated with primary antibody in blocking solution for overnight at 4°C. The membranes were then washed in TBST and incubated with secondary antibody conjugated to horseradish peroxidase for 1 h at room temperature. After the incubation, the membranes were washed with TBST, and immunoreactive bands were detected using an enhanced chemiluminescence solution kit (PerkinElmer Life Sciences).

### DiI labeling of dendrites and spine analysis

P10 mice were anesthetized and quickly perfused with 4 ml of PBS followed by 10 ml of 4% PFA in PBS solution. Mice where decapitated, brains were removed and further fixed for 10 min in 4% PFA, then moved to cold PBS. Hippocampi were removed and the tissue chopper was used to section hippocampi into 100-µm slices. Slices were labeled using 1,1'-dioctadecyl-3,3,3',3'-tetramethylindocarbocyanine perchlorate (DiI)-coated tungsten particles as described in Gan et al. (2000). Images were taken using a spinning disk microscope at 63× (0.3-µm step size) of entire dendrite sections. Sections of apical dendrites were selected from the primary branch point between 25 and 40 µm in length mean values were obtained from four to six slices, and the mean values per animal were considered an *N*. Analysis was performed on max projections of z-stacks and measured in ImageJ. Measurements of spine length (L), head width (Hw), and neck width (Nw) were taken. Spine types were categorized as mushroom (ratio Hw:Nw > 1.5), stubby (ratio Hw:Nw = 0.5-1.5 and L:Nw < 1.5), and thin (ratio Hw:Nw = 0.5-1.5 and L:Nw > 1.5). Genotypes were blinded to the observer during labeling, imaging, and analyses.

### GST-rhotekin binding domain (RBD) pulldown assay to measure active RhoA

To detect active GTP-bound RhoA in lysates, lysates were prepared from high density (500,000 cells per well on a six-well plate) cortical neuron cultures prepared as described above/below and grown for 15 DIV. Cells were washed in ice cold TBS 10 mM Tris (pH 7.5) and 150 mM NaCl) and lysed in Mg^2+^ lysis buffer [25 mM HEPES (pH 7.5), 150 mM NaCl, 1% NP-40, 10 mM MgCl_2_, 1 mM EDTA, 10% glycerol, with inhibitor mix (aprotinin (0.5 μg/ml), leupeptin (0.5 μg/ml), benzamidine (100 μg/ml), and PMSF (20 μg/ml) and phosphatase inhibitors 25 mM NaF, 1 mM NaOV_4_. For a positive control, 20 µl of 0.5 M EDTA and 10 µl of GTPγS were added to one lysate and incubated at 30°C for 30 min, and the reaction was quenched with 60 mM MgC1_2_. Lysates were incubated with GST-RBD beads for 1 h at 4°C. GST-RBD-bound proteins were eluted in Lammeli sample buffer and analyzed by Western blotting. GST-RBD beads were prepared as originally described by Ren et al. (1999). Briefly, pGEX2T-RBD transformed *Escherichia Coli*, were stimulated with 1 mM IPTG at 30°C. Pelleted cells were resuspended in 20 mM HEPES (pH 7.5), 150 mM NaCl, and 5 mM MgCl_2_ with protease inhibitor mix. Cells were lysed by repeated freezing and thawing, followed by sonication and addition of Triton X-100, to a final concentration of 0.1%. Lysates were incubated with GST beads for 1 h, washed with lysis buffer, and eluted in Laemmli sample buffer, then visualize by Western blotting. Samples were analyzed by Western blotting for RhoA (Millipore, 05-778, RRID:AB_11213369).

### COS7 cell spreading/contraction assay

COS7 cells (RRID:CVCL_0224) were maintained in DMEM with 10% bovine calf serum, 2 mM L-glutamine, and 100 μg/ml P/S. For transfection, 50,000 cells were seeded 1 d before transfection in 12-well plates. The following day, cells were transfected with a total of 2 μg of DNA using lipofectamine 2000 (Invitrogen)_._ DNA and lipofectamine were separately suspended in Opti-MEM media and then mixed. COS7 were washed in media without serum, and then the transection mix was added to the cells. Forty-eight hours after transfection, COS7 were trypsinized and reseeded onto glass coverslips (12 mm, Fisher Scientific), coated with laminin (0.5 μg/ml) for 2 h, at a density of 5000 cells. Cells were fixed in 4% PFA in PBS for 30 min at room temperature 24 h after seeding and then washed with PBS and incubated with rhodamine-tagged WGA (5 μg/ml) in PBS for 10 min. Coverslips were washed with PBS (3 × 5 min) and then quickly with water before mounting with anti-fading mounting media (Dako). Imaging was performed using a 40× objective on a Zeiss Axioskop fluorescent inverted microscope equipped with Xenon illumination, and images were captured using Zen software (Zeiss, RRID:SCR_014587). Cells too large to fit in one field of view and cells that appeared as bright rounded balls without any cytoplasm were excluded from analysis. The cell area of GFP-expressing cells was quantified using NIH ImageJ software (Fiji, RRID:SCR_002285) as follows. Images of rhodamine-tagged WGA membrane labeling were thresholded and made into mask and added as a ROI. ROIs were added to the GFP image and ROIs that were not GFP positive were deleted. The area of each cell (ROI) was determined using the measure tool.

### Experimental design and statistical analysis

Statistical analysis was done using GraphPad Prism 6.0 (GraphPad Software, RRID:SCR_002798). Number of replicate and statistical tests are specified in figure legend for each experiment.

## Results

### LGI1 regulates hippocampal neuron synapse number *in vitro*

**Figure 1.**
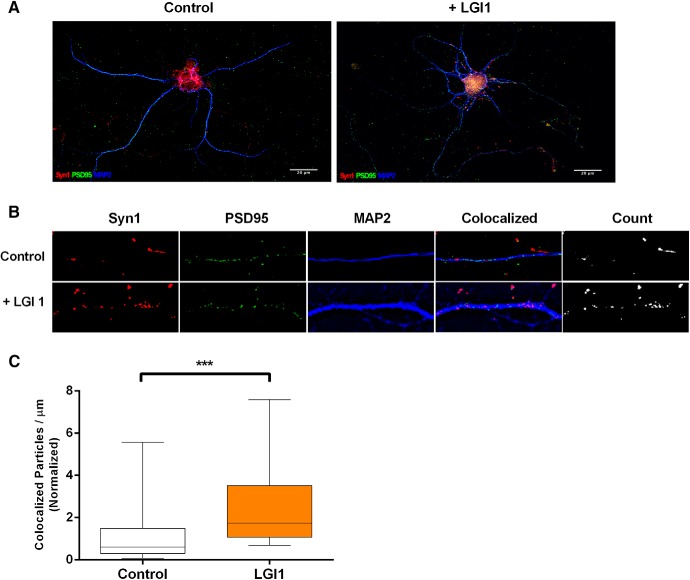
Exogenous application of LGI1 promotes synapse formation. ***A***, Representative images from 18 DIV cultures hippocampal neuron labeled with Syn1 (red), PSD95 (green), and MAP2 (blue) in the absence and presence of exogenous LGI1 (6 DIV treatment). ***B***, Magnification of sample images; the panel labeled “count” indicates colocalized particles of Syn1 and PSD95 considered to be synaptic puncta. ***C***, Quantification of synaptic density indicated by colocalized puncta per µm of MAP2-labeled dendrites excluding the cell bodies. Data are shown as box plots with whiskers from 1^st^ to 99th percentile. Differences were analyzed by unpaired *t* tests, ***p = 0.0007; *N* = 34 WT and 29 treatment neurons from three separate cultures (8–14 images were included for each condition in each experiment).

NgR1 activity suppresses synapse formation in the CNS and LGI1 acts as an NgR1 antagonist. Therefore, we hypothesized that during development, LGI1 functions to “suppress the suppressor,” a prediction of this model is that application of exogenous LGI1 should favor synapse formation. To address this, WT hippocampal neurons were cultured *in vitro* for 13 d then subjected to either normal media or to media supplemented with purified LGI1 for 6 d and then fixed at 18 DIV (final LGI1 concentration was 250 nM). After treatment cells were fix and immunocytochemistry was used to detect Syn1 and PSD95, the pre- and postsynaptic markers used to quantify synapse number, as detailed in Materials and Methods. Interestingly, treatment with exogenous LGI1 caused a large increase in synaptic density, enhancing synapse number from a normalized baseline of 1.00 to 2.49 (Fig. 1*A–C*). Thus, exogenous LGI1 potently promotes synapse formation on hippocampal neurons *in vitro*.

**Figure 2.**
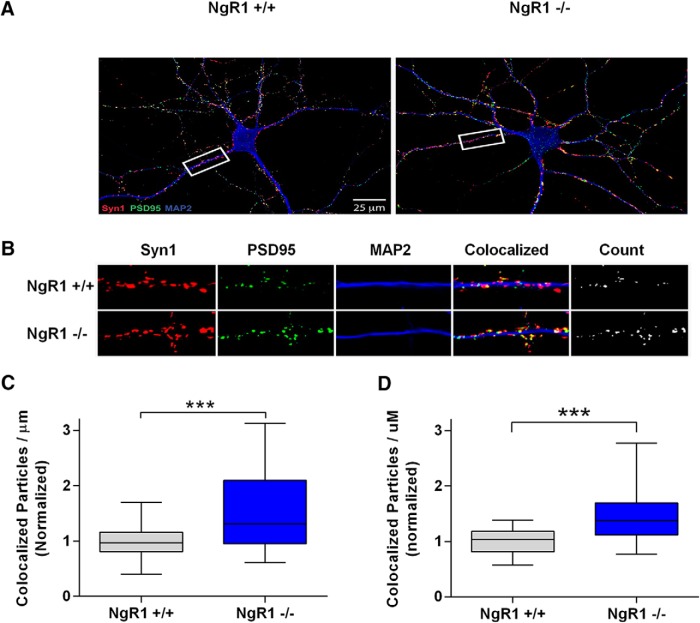
NgR1 regulates synapse number in dissociated hippocampal neurons. Analysis of synapse density from cultured hippocampal neurons grown on coverslips for 15 or 18 DIV. ***A***, Representative images from 18 DIV cultures labeled with Syn1 (red), PSD95 (green), and MAP2 (blue), genotypes are indicated. ***B***, Magnification of sample images shown in the white box in ***A*** used for quantification. The panel labeled “count” indicates colocalized particles of Syn1 and PSD95 considered to be synaptic puncta. ***C***, Quantification of synaptic density indicated by colocalized puncta per µm of MAP2-labeled dendrites excluding the cell bodies for 15 DIV cultures. ***D***, Same as ***C*** but for 18 DIV cultures. Values in quantification were normalized to WT littermates within each culture. Data are shown as Box plots with whiskers from 1st to 99th percentile. NgR1^-/-^ neurons display significantly more synapses than NgR1^+/+^ littermates. For 15 DIV cultures, NgR1^+/+^ *n* = 29 neurons and NgR1^-/-^ *n* = 28 neurons from three cultures. For 18 DIV cultures, NgR1^+/+^ *n* = 25 neurons and NgR1^-/-^ *n* = 31 neurons from three separate cultures. Differences were analyzed by unpaired *t* tests, ***p < 0.001.

**Figure 3.**
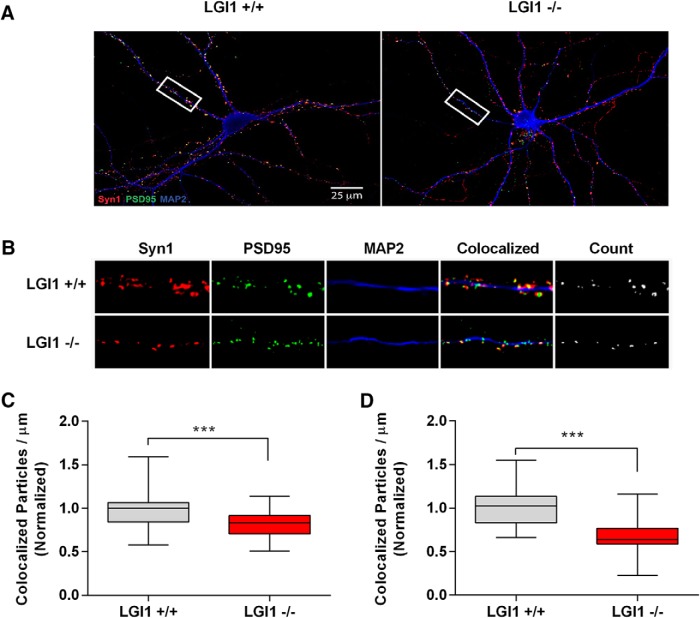
LGI1 regulates synapse number in dissociated hippocampal neurons. Analysis of synapse density from hippocampal neurons dissociated from E16 embryos grown on coverslips for 15 or 18 DIV. Panels ***A***, ***B*** show representative images from 18 DIV cultures labeled with Syn1 (red), PSD95 (green), and MAP2 (blue), genotypes are indicated. Sample images of LGI1^+/+^ and LGI1^-/-^ neurons used in analysis of synaptic density, quantified from the number of colocalized Syn1 and PSD95 puncta per μm of MAP2-labeled dendrites excluding the cell bodies. ***B***, Enlarged images from the box highlighted in ***A***. The far right panel indicated “count” shows the colocalized puncta representing the synapse. Values in quantification were normalized to WT littermates within each culture. Data are shown as box plots with whiskers from 1st to 99th percentile. LGI1^-/-^ neurons have significantly fewer synapses than LGI1^+/+^ littermates. ***C***, Synapse density quantified from 15 DIV cultures. ***D***, Synapse density quantified from 18 DIV cultures. LGI1^+/+^ *n* = 40 neurons from four cultures, LGI1^-/-^ *n* = 40 from four cultures; ***p < 0.001 (unpaired *t* test).

A key aspect of our model is that NgR1 activity reduces synapse number in hippocampal neurons, as reported previously (Wills et al., 2012). To confirm this, we analyzed synapse number in hippocampal neurons derived from NgR1^-/-^ mice or their WT littermates at two time points: 15 DIV approaching the peak of *in vitro* synapse formation and 18 DIV the point at which cultures are first considered mature. Figure 2*A–D* shows that NgR1^-/-^ hippocampal neurons have significantly more synapses than their WT counterparts, both at 15 DIV (increased 52%, normalized values increased to 1.523), and at 18 DIV (increased 47% normalized values increased to 1.468), agreeing with findings of Wills et al. (2012). Given this effect of NgR1, and given our previous findings showing that LGI1 normally acts to suppress NgR1 activity (Thomas et al., 2010), we predicted that loss of exogenous LGI1 would result in increased NgR1 activity that would in turn reduce synapse number. To address the latter point, we assessed synapse number in hippocampal neurons derived from LGI1^-/-^ mice or their WT littermates at two time points. Figure 3*A–D* shows that the number of synapses was significantly decreased in LGI1^-/-^ neurons examined at 15 DIV (reduced 18%, normalize values decreased from 1-0.82) and at 18 DIV (reduced 33%, normalized values decreased from 1 to 0.66). Thus, loss of exogenous NgR1 and loss of LGI1 produced qualitative results that are consistent with our model.

**Figure 4.**
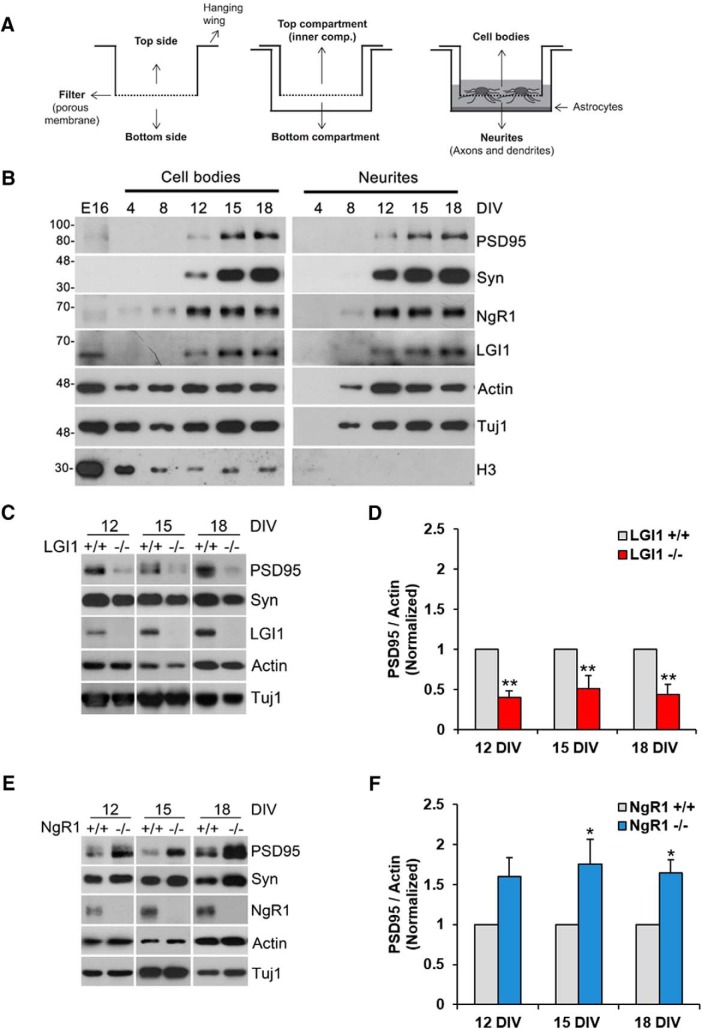
NgR1 and LGI1 regulate synaptic proteins in cortical neurons *in vitro.* ***A***, Twiss filter schematic showing culture system to coculture hippocampal neurons with astrocytes and separate neuronal processes from cell bodies. Hippocampal neurons seeded on filters with a pore size 1 µm that cell bodies will not pass through. Axons and dendrites grow on the filter tops and extend down onto the filter bottom. Astrocytes are seeded on the bottom of the well to provide growth factors. ***B***, Time course of lysates from hippocampal neurons grown on filters suspended over an astrocyte feeder layer for the times indicated. The first lane in the left panel labeled E16 is a sample of hippocampal neurons lysed directly after dissociated before plating. Lysates from filter tops including cell bodies and processes are on the left. Lysates of the filter bottoms containing axons and dendrites but no cell bodies are on the right. Antibodies used to probe the lysates are indicated on the right. Histone-3 (H3), a structural protein found in chromatin and present only in the nucleus is detected only in the cell body lysates. ***C***, Lysates from filter bottoms containing axons and dendrite but not cell bodies from LGI1^+/+^ and LGI1^-/-^ littermates of cortical cultures grown for the indicated number of DIV. ***D***, Quantification of PSD95 levels relative to actin levels and normalized to WT controls in LGI1 samples at 12, 15, and 18 DIV, *n* = 3 separate experiments. ***E***, Western blottings of lysates from filter bottoms of NgR1^+/+^ and NgR1^-/-^ cortical cultures harvested at 12, 15, or 18 DIV synaptic markers, Syn and PSD95. Actin and Tuj1 are loading controls. ***F***, Quantification of PSD95 relative to actin levels and normalized to WT controls in NgR1, *n* = 4 separate experiments. Significant differences are indicated on the graphs analysis was performed by two-way ANOVA with Bonferroni *post hoc* tests, **p < 0.01, *p < 0.05.

To gain insight into the role of NgR1 and LGI1 on the developmental progression of synapse formation *in vitro*, we developed a filter-based assay that allowed us to monitor the accumulation of synaptic marker proteins selectively in neurites undergoing synaptogenesis (Unsain et al., 2014). In this approach, primary hippocampal neurons were plated on the top of a filter insert and maintained in wells containing an astrocyte feeder layer (shown schematically in Fig. 4*A*). Over several days, neurites project from the cell body and a subset of these project to the bottom of the filter, where they eventually make contacts with neighboring neurites and form synapses. The cell bodies are too large to pass through the filter pores and are therefore retained on the filter top. Thus, by collecting material from the filter bottom, we can assess the selective accumulation of synaptic markers in the neurite compartment.

Figure 4*B* shows the accumulation of several proteins on the top and bottom of the filters from WT neurons maintained 4, 8, 12, 15, and 18 DIV. Histone H3 was present only on the filter top, indicating that cell bodies are retained in this compartment (Fig. 4*B*, bottom lane). In the top compartment, levels of actin and tubulin levels remain relatively steady whereas levels of Syn and PSD95 become detectable and rise dramatically starting at 12 DIV (Fig. 4*B*). Levels of LGI1 and NgR1 also start to increase in the top compartment at 12 DIV, consistent with their playing a role in the regulation of synapse formation. In the bottom compartment, actin and tubulin become detectable at 8 DIV and increase until 12 DIV, likely reflecting the growth of neurites onto the bottom of the filter. Syn, PSD95, LGI1, and NgR1 are undetectable at 8 DIV but readily detectable at 12 DIV, after which they remain stable.

With these baselines established, we then examined the protein composition of LGI1^-/-^ and NgR1^-/-^ neurites projecting to the bottom cell-free compartment, relative to WT littermates in each instance. Figure 4*C*,*D* shows PSD95 levels are sharply decreased (*p* = 3.69e-05), and Syn levels modestly and not significantly decreased (*p* = 0.0807), in LGI1^-/-^ neurites examined at 12, 15, or 18 DIV. In direct contrast, NgR1^-/-^ mice examined at 12, 15, and 18 DIV show a strong increase in PSD95 levels (*p* = 0.00024), as well as a more modest and not significant, increase in Syn levels (*p* = 0.0978; Fig. 4*E*,*F*). Taken together with other findings above, these results suggest that NgR1 suppresses synaptogenesis and that LGI1 antagonizes this effect.

**Figure 5.**
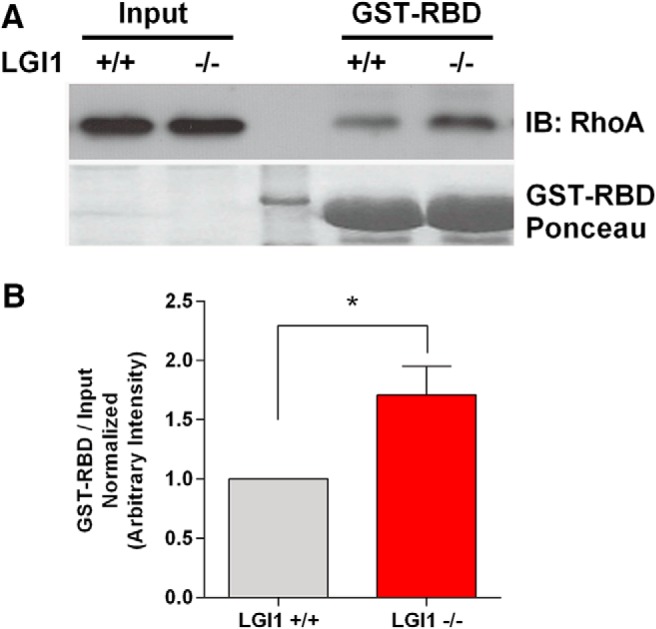
LGI1 normally suppresses RhoA activity in cultured cortical neurons. A total of 15 DIV cortical neurons from LGI1^-/-^ mice have increased RhoA activation compared to WT cultures. ***A***, Western blottings of input RhoA and active RhoA bound to GST-rhotekin beads (GST-RBD) that bind to GTP-RhoA. The lower panel corresponds to the membrane labeled with Ponceau showing total GST protein. ***B***, Quantification of active GTP-RhoA relative to total RhoA input levels and normalized WT values for each of four experiments. Active GTP-RhoA is significantly higher in lysates from LGI1^-/-^ cortical neurons compared to LGI1^+/+^ controls, 71% higher, *p* = 0.0257, analyzed by unpaired *t* test; *N* = 4 separate experiments.

**Figure 6.**
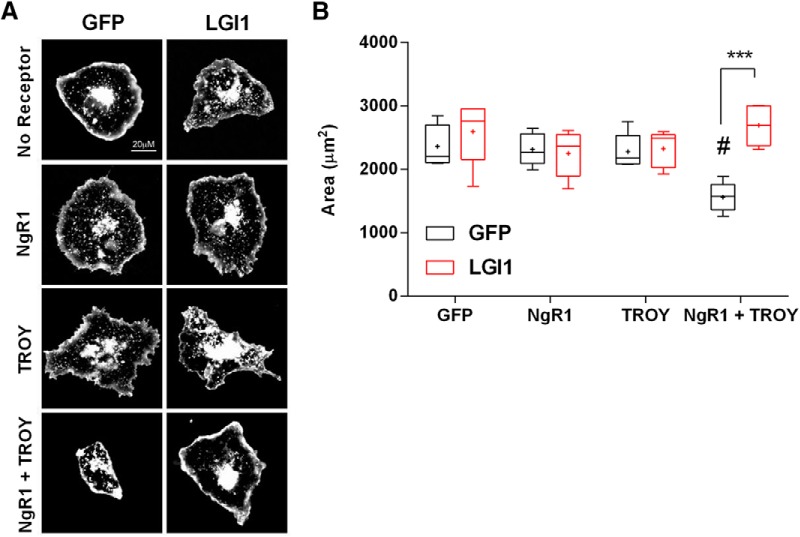
LGI1 regulates a NgR1-TROY receptor complex. ***A***, Sample images of COS7 cells transfected with control GFP only; NgR1; TROY; NgR1 + TROY: LGI1; NgR1 + LGI1; TROY + LGI1; NgR1 + TROY + LGI1, together labeled with rhodamine-WGA. COS7 cells transfected with the indicated plasmids and seeded at low density on glass coverslips. In all conditions GFP was cotransfected to follow the expression of the other proteins. Cell membranes were labeled with rhodamine-tagged WGA to visualize cells and measure cell area. ***B***, Area of the cells was quantified using ImageJ Analyze Particles tools. The graph indicates the effect of expression of no receptors (GFP control), each receptor alone, or with LGI1 (indicated in red). Expression of LGI1, NgR1, or TROY separately in COS7 cells has no effect on cell size compared to GFP alone. However, NgR1 and TROY expression together decrease cell size compared to GFP expression alone, *p* < 0.05 indicated by # on the graph. Coexpression of LGI1 together with NgR1 and TROY rescues cell size. This condition is not significantly different from GFP alone. Analysis by two-way ANOVA followed by Bonferroni *post hoc* tests, ***p < 0.001; *n* = 5 where each n is the average area in one separate experiment. For each experiment the areas of 50–100 cells were quantified.

By physically associating with transmembrane proteins, p75NTR or Troy, NgR1 forms the ligand-binding portion of a receptor complex that induces RhoA activity. Since LGI1 can function as a NgR1 antagonist, we predicted that loss of endogenous LGI1 would increase constitutive RhoA activation levels in primary central neurons. To address this, LGI1^-/-^ cortical neurons, and corresponding littermate WT neurons, were maintained *in vitro* for 15 d and then subjected to GST-rhotekin pulldown assays to assess active RhoA levels. Figure 5*A*,*B* shows that levels of active RhoA are 71% higher in LGI1^-/-^ primary neurons than in their WT counterparts, consistent with the hypothesis that endogenous LGI1 suppresses constitutive NgR1-dependent RhoA activity in cortical neurons.p75NTR levels are very low in the hippocampus and the NgR1 signaling complex consists of NgR1 complexed with TROY (Wills et al., 2012). To determine whether LGI1 can alter signaling mediated by the NgR1-TROY complex, we developed a reconstituted cellular assay that would allow us to assess the role of individual components in the signaling cascade, in the absence of potential compensatory receptors and ligands. The approach is based on the RhoA-dependent contraction in cell size that occurs in COS7 cells and allows us to manipulate specific signaling components and assess their effects on RhoA activation, measuring cell area as a proxy for small GTPase activation (Zeinieh et al., 2015; Thomas et al., 2016). It is well established RhoA mediates cell contraction, by initiating actin cytoskeleton remodeling (for review, see Kaibuchi et al., 1999; Etienne-Manneville and Hall, 2002) and that NgR1 together with a coreceptor activates RhoA signaling (Wong et al., 2002; Yamashita and Tohyama, 2003; Park et al., 2005; Shao et al., 2005; Wills et al., 2012). Figure 6*A*,*B* shows that when expressed individually, neither LGI1, NgR1, or TROY had any effect on cell size. However, coexpression of NgR1 and TROY causes a highly significant cellular contraction, suggesting that they interact and activate RhoA in COS7 cells. Figure 6*B* shows that this RhoA-dependent contraction was completely blocked when LGI1 was coexpressed with NgR1 and TROY, indicating that LGI1 is a potent antagonist of the NgR1-TROY complex.

### LGI1 deletion results in changes in spine morphology and synaptic function

Our next series of studies examined the effects of LGI1 on synaptic morphology and function *in vivo*. In these studies, we had to balance the complications of assessing synapse structure and function in juvenile animals against the incipient seizures that are initiated in the perinatal period, at P11. We therefore chose P10 as the most appropriate age in which to carry out these studies.

**Figure 7.**
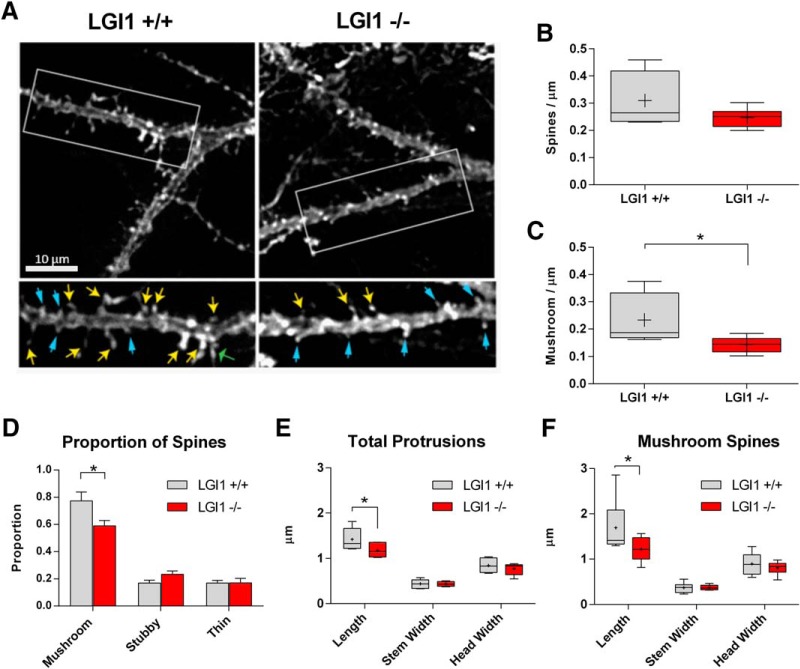
Dendritic spine density and morphology is regulated by LGI1 *in vivo*. ***A***, Sample images of dendritic spines labeled with DiI from acute hippocampal slices from P10 LGI1^+/+^ (left) and LGI1^-/-^ (right) mice. Segments were taken from the primary bifurcation point from CA1 pyramidal neurons shown in the upper images. The selected region if dendrite is shown below with arrows indicating mushroom spines (yellow), stubby spines (blue, thin arrowheads), and thin spines (green, wide arrowheads). Images were manually traced using the measure tool in ImageJ. ***B***, Quantification of total spine-like protrusion density in spines per µm of dendrite. There is a non-significant trend toward fewer spine-link protrusions in LGI1^-/-^ mice and WT controls (unpaired *t* test, *p* = 0.17). ***C***, Proportion of spines categorized based on ratios of the width of the spine head (Hw) to width of the spine neck (Nw) to the spine length, mushroom spines (Hw:Nw > 1.5), stubby spines (Hw:Nw < 1.5 and L:Nw < 1.5), and thin spines (Hw:Nw < 1.5 and L:Nw > 1.5). The proportion of mushroom spines is significantly less in LGI1^-/-^ slices compared to WT controls (*p* = 0.03), stubby spines trend toward an increase (*p* = 0.08), and the proportion of thin spines is unchanged, analyzed by two-way ANOVA with Bonferroni *post hoc* tests. ***D***, Quantification of mushroom type protrusions per µm of dendrite. The density of mushroom type spines is significantly decreased in LGI1^-/-^ slices compared to WT controls, compared by Student’s *t* test (*p = 0.04). ***E***, Measurements of spine length, neck width, and head width for total spines, significant differences indicated on the graph (*p = 0.031), analyzed by two-way ANOVA with Bonferroni *post hoc* tests. ***F***, Measurements of spine length, neck width, and head width for mushroom type spines, significant differences indicated on the graph (*p = 0.034) analyzed by two-way ANOVA with Bonferroni *post hoc* tests. For spine analysis *n* = 6 animals for each genotype, littermates from three separate experiments, four to six spine segments per animal were quantified.

To analyze dendritic spine morphology *in vivo*, we generated hippocampal slices from P10 LGI1^-/-^ and littermate mice and used a biolistic approach to label well separated CA1 pyramidal neurons with DiI. Spine length, neck width and head width were measured on 25- to 40-µm sections of apical dendrites, just distal to the primary branch point, which allowed us to categorize spines as mushroom, stubby, or thin. Figure 7*A* shows representative images of the segments used for analysis. The number of total spines showed a trend toward being reduced in LGI1^-/-^ mice compared to WT neurons (*p* = 0.17; Fig. 7*B*) . Interestingly, there was a significant reduction in the incidence of mushroom spines (Fig. 7*C*). We observed a significant decrease in the proportion of mushroom spines, but the corresponding increase in other spine types was not significant (Fig. 7*D*). We also analyzed the spine length, spine stem width, and spine head width across all categories of spines and found that overall spine length was significantly decreased in LGI1^-/-^ mice compared to controls (Fig. 7*E*). Focusing specifically on mushroom-like spines, we found that LGI1^-/-^ neurons displayed a significant decrease in spines length compared to WT littermates (Fig. 7*F*). Taken together, these data indicate that P10 LGI1^-/-^ mice display a reduction in the number and length of mushroom-type dendritic spines.

**Figure 8.**
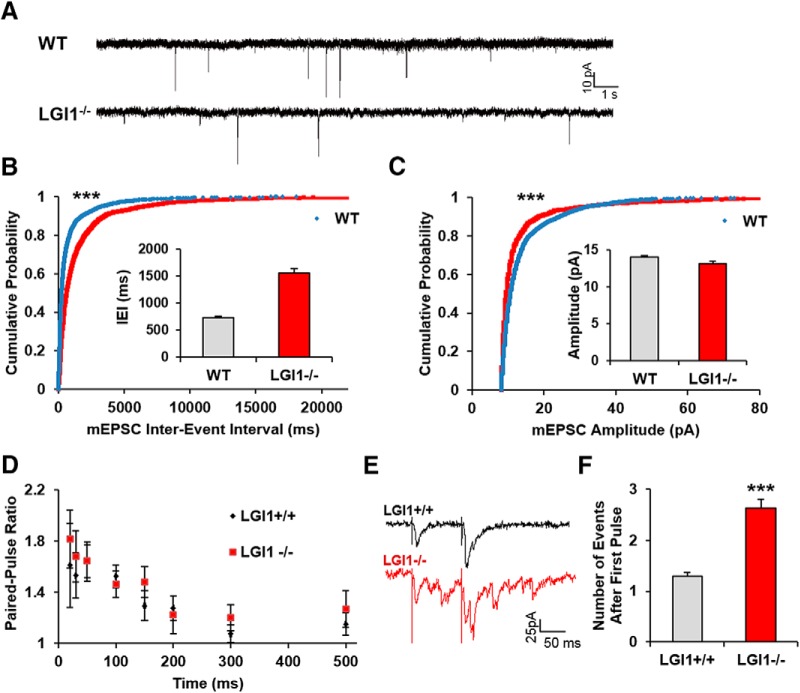
LGI1 deletion alters synaptic activity in the hippocampus. Recordings of mEPSC of CA1 pyramidal neurons in acute hippocampal slices from P10 (A-C) LGI1^-/-^, and LGI1^+/+^ littermates. ***A***, Representative traces of mEPSCs. ***B***, Cumulative probability plot of interevent intervals with inserts showing average values and SE bars. ***C***, Cumulative probability graph of mEPSC amplitude, with inserts showing average values and SE bars. Statistical comparisons for mEPSCs were made from cumulative probability data using the Kolmogorov–Smirnov test, ***p < 0.001; *n* > 2000 events from 7 to 12 cells from three to four separate animals. ***D***, Paired-pulse ratio measurement; quantification of the ratio of eEPSC amplitude between the first and second pulse, WT *n* = 11 cells, LGI1^-/-^ *n* = 12 cells. We found no significant differences between pulses, analyzed by *t* test. ***E***, Sample traces of paired-pulse facilitation of CA3-CA1 with 100 ms between pulses. ***F***, Quantification of eEPSC in CA1 neurons after the first stimulation of Schaffer collaterals in the CA3 *n* = 60 traces for WT and *n* = 59 traces for LGI1^-/-^ condition ***p < 0.0001 (*t* test).

To determine whether the changes in spine morphology correlate with alterations in synaptic activity, we measured mEPSCs in P10 hippocampal CA1 neurons in acute slices derived from LGI1^-/-^ and WT littermates. Resting membrane potential input resistance and input-output curves did not differ between LGI1^-/-^ and their WT littermates. However, LGI1^-/-^ mice displayed a significant increase in mEPSC interevent interval compared to WT littermate controls (Fig. 8*A*,*B*). Taken together with the morphologic data above, this finding suggests that P10 LGI1^-/-^ mice may have weaker synapses that their WT controls. Consistent with this, we found that mEPSC amplitude was modestly but significantly decreased in the CA1 region of LGI1^-/-^ null mice (14.01 ± 0.18 pA in WT vs 13.12 ± 0.33 pA in LGI1^-/-^; Fig. 8*C*).

To test whether the changes in LGI1^-/-^ mice reflected a deficit in presynaptic transmission, we assessed paired-pulse facilitation of CA3 to CA1 synapses. When the lag between first and second paired stimuli ranged from 20 to 500 ms, the ratio of the evoked amplitude did not differ between LGI1^-/-^ versus WT, indicating that at least by this measure, presynaptic transmission is normal in LGI1^-/-^ mice (Fig. 8*D*). Intriguingly, although P10 mice do not undergo frank seizures, we observed that LGI1^-/-^ neurons consistently displayed a large increase in event frequency after the first paired stimulus, suggesting that this activity evokes an epileptiform-like discharge (Fig. 8*E*,*F*). Taken together, the morphologic and electrophysiological results indicate that P10 LGI1^-/-^ animals have weaker synapses than their WT counterparts and that they exist in a pre-epileptic state in which spontaneous activity is reduced but evoked activity is readily induced.

## Discussion

In the present study, we provide the first evidence that LGI1 directly promotes synapse formation.

Previous result showing that NgR1 activity suppresses synapse formation (Wills et al., 2012; Akbik et al., 2013) and that LGI1 antagonize NgR1 activity (Thomas et al., 2010) prompted the hypothesis that LGI1 facilitates synapse formation. We observed that the addition of exogenous LGI1 to primary cultures of hippocampal neurons induced a 75% increase in synapse number. The striking result led us to investigate if hippocampal neurons from LGI1 null mice showed defects in synapse formation. We found a highly significant decrease in synapse number in neuronal cultures from LGI1^-/-^ mice compared to WT control cultures. Observing that exogenous acute application of LGI1 increases synapse formation and knockout of LGI1 reduces synapse formation *in vivo* has led us to conclude that LGI1 is a physiologic regulator of synapse formation.

We previously found LGI1 antagonizes NgR1 signaling in the context of neurite outgrowth on myelin and thus predicted that LGI1 would also act antagonistically to NgR1 in the context of synapse formation. to investigate the relationship between LGI1 and NgR1 in synapse formation, we quantified synapses in cultures of hippocampal neurons from NgR1^-/-^ mice. In line with our results on LGI1^-/-^ neurons, and consistent with the findings of Wills et al. (2012), we found that NgR1^-/-^ hippocampal neurons have ∼50% more synapses than their WT counterparts. We propose the increase in synapse number seen after addition of exogenous LGI1 represents at least in part the antagonism of the NgR1 mediate synapse repression or elimination. It is conceivable that LGI1 may also be promoting synapse formation directly through another pathway, possibly by stabilizing synapses via ADAM22/23 (Lovero et al., 2015) or regulating potassium channel subcellular localization (Boillot et al., 2016). However, we found that the protein expression level of ADAM22 in COS7 cells is below the immunoblot detection limit (data not shown) suggesting that the role of LGI1 is independent of ADAM22 in this context. Nevertheless, the contributions and interactions between different LGI1 signaling pathways remains an interesting avenue for future studies. Primary cultures of hippocampal neurons develop synapses after several days of culture, generally around 12–14 DIV. To further describe of the role of NgR1 and LGI1 in our cultures of hippocampal neurons, we followed the expression of synaptic markers, as well as NgR1 and LGI1, at various time points after seeding the neurons. We tested the hypothesis that the expression of LGI1 and NgR1 follow the expression profile of the synaptic markers PSD95 and Syn1. Indeed, we observe that the expression of LGI1 and NgR1 increased concurrently with the appearance of synaptic markers in the axonal/dendritic compartment of hippocampal neurons grown on filters. We then asked if LGI1^-/-^ and NgR1^-/-^ neurons show a different pattern of expression of synaptic markers than control WT neurons. In agreement with our data on synapse formation describe above, we found that the levels of synaptic markers are decreased in the absence of LGI1 and increased in the absence of NgR1. Thus, our *in vitro* analyses have revealed that NgR1 preventing synapses formation whereas LGI1 promotes synapses formation.

We then sought to find a mechanism by which LGI1 can promote synapse formation. It is well described that RhoA regulates synaptogenesis by controlling the actin cytoskeleton (Duman et al., 2015) and that NgR1 is a potent regulator of RhoA signaling (Yamashita and Tohyama, 2003; Wills et al., 2012). We hypothesized that LGI1 acts to reduce NgR1-mediated activation of RhoA and predicted that level of RhoA activity will be increased in neurons lacking LGI1. Using a well characterized method to assess RhoA activity, we provide direct evidence that RhoA activity is strongly increased in neurons lacking LGI1. To investigate whether LGI1 actually regulates RhoA activity through the NgR1 receptor, we used a well-validated reconstituted system (Zeinieh et al., 2015), in which receptor and ligand expression can be controlled and cell size contraction acts as an accurate read-out for RhoA activity. This approach showed that NgR1 and TROY coexpression dramatically increased RhoA activity and that coexpression of LGI1 strongly counteracts this effect. Taken together, our data show that LGI1 facilitates synapse formation by antagonizing NgR1-dependent RhoA activity.

To characterize the role of LGI1 in synapse formation *in vivo*, we investigated the effect of LGI1 deletion in synapse morphology and synaptic activity in brain slices. The total number of spines was not affected but mushroom spine number was significantly reduced in LGI1 null mice. These structural changes are consistent with a recent electron microscopic analysis of the cortical region in a distinct LGI1^-/-^ null strain, which reported fewer active asymmetric axospinal synapses in LGI1 null animals (Silva et al., 2015). These authors also observed a decrease in spine number and shorter spines within the motor cortex of LGI1 null animals, consistent with our findings in hippocampal neurons.

These morphologic results suggested that LGI1 must be present for synapses to reach maturity, spurring us to examine synaptic transmission in hippocampus lacking LGI1 expression. Whole cell patch clamp recording in CA1 pyramidal neurons showed that mEPSC amplitude is significantly reduced, and mEPSC interevent interval was increased in LGI1^-/-^ brain slices. The electrophysiological results suggest that synapses in LGI1^-/-^ mice are weaker or less abundant in comparison to WT mice. Together, our findings strongly support the hypothesis that LGI1 promotes synapse formation and maturation, *in vitro* and *in vivo*.

Interestingly, although we did not observe seizures in mice at the time of the hippocampal preparation (P10), we did observe epileptic activity in P10 hippocampal slices from LGI1^-/-^ mice after evoked EPSC. We observed this activity using a paired pulse facilitation paradigm whereas an earlier study by Yu et al., detected epileptiform activity in field potentials of LGI1^-/-^ slices exposed to Mg^2+^-free media (Yu et al., 2010). The mechanisms that account for epileptogenic activity in LGI1^-/-^ preparations are not known, but it is notable that basal and evoked IPSCs seem normal in LGI1 null hippocampal slices (Fukata et al., 2010; Yu et al., 2010). It is also interesting that selective deletion of LGI1 from glutamatergic neurons generates spontaneous epileptic behavior in mice, whereas mice lacking GABAergic interneuron expression of LGI1 appear normal (Boillot et al., 2014), indicating that LGI1 expression in excitatory neurons is required to suppress epileptogenic behavior. It is also notable that deletion of LGI1 has shown to decrease the expression of the potassium voltage-gated channel Kv1.1 (Seagar et al., 2017), perhaps facilitating run-away currents in these developing networks. It is conceivable that the decrease in the number of excitatory synapses could produce a homeostatic response that result in a lower of threshold for firing after stimulation, such that stimuli that normally produce a single evoked response result in epileptic activity.

RhoA is known to play a key role in regulating neuronal morphology during development, synapse formation, spine dynamics, and synaptic plasticity (Luo, 2000; Hering and Sheng, 2001). Growing evidence suggests dysregulation of RhoA signaling contributes to the etiology of multiple psychiatric disorders, including schizophrenia (Hill et al., 2006). Significantly, mutations in NgR1 have been associated with schizophrenia (Budel et al., 2008) and we have recently identified mutations in NgR1 and LGI1 within schizophrenia patients that cause impairments in NgR1-LGI1-RhoA signaling (Thomas et al., 2016). Thus, we believe that understanding the details of LGI1-NgR1-RhoA regulation in different signaling contexts may be important for designing novel therapeutics.

Although many molecules have been identified that promote synapse formation and maturation, fewer molecules are known to regulate synapse removal and turnover. Here we provide support for the hypothesis that a coregulatory loop involving NgR1 and LGI1 is an important regulator of synaptic structure within excitatory neurons of the hippocampus. The correlation of LGI1 and NgR1 levels with synapse numbers shown in this study suggests that LGI1 normally plays a critical role in regulating synaptic stability and elimination.

## Synthesis

Reviewing Editor: Pablo Castillo, Albert Einstein College of Medicine Decisions are customarily a result of the Reviewing Editor and the peer reviewers coming together and discussing their recommendations until a consensus is reached. When revisions are invited, a fact-based synthesis statement explaining their decision and outlining what is needed to prepare a revision will be listed below. The following reviewer(s) agreed to reveal their identity: Marta Zagrebelsky. Note: If this manuscript was transferred from JNeurosci and a decision was made to accept the manuscript without peer review, a brief statement to this effect will instead be what is listed below.

Your manuscript has been evaluated by two experts in the field. They both agree on the quality and significance of your results. The reviewers understand your study provides good evidence that LGI1 gain and loss-of-function modulates excitatory synapse assembly in primary hippocampal neurons. However, they also raised a number of concerns that you should properly address before your paper can be considered for publication. Here, I summarize some points of agreement from their online discussion:

1. The authors cannot conclude neuronal exposure to LGI1 peptide specifically promotes synapse formation, since increased synapse density in response to LGI1 could be the result of an inhibition of synapse elimination. The authors should modify their writing and discuss this alternative possibility.

2. The paper would be strengthened by directly implicating LGI1 with NgR1 and/ or Adam 22, since the authors provide no direct evidence linking LGI1 with these regulators of synpatogenesis, only indirect evidence that neuron cultures from LGI1 knockouts show augmented RhoA activity, a mediator of NgR1 signaling as well as other regulators of synaptogenesis. The link between LGI1 and NgR1 in coregulating synaptogenesis is only correlative at this point. The authors should highlight this limitation in the discussion.

3. The dendritic spine images provided in Figure 8 are not representative of the quantification provided. Given this analysis is from P10 animals, a relatively early stage of synaptogenesis, the low spine density is not surprising; however, the width and intensity (DiI staining) of the dendritic segments provided for each condition in Figure 8 are not equivalent, complicating the interpretation of the data. The authors should provide better representative images including the whole neuron with the apical dendritic segment chosen (boxed) for each condition and indicate which spines are mushroom type (arrows).

In addition to these three main points, you should address all other points raised by the reviewers in their specific comments (see below).

---------------------------------------------------------------

REVIEWER 1

In the manuscript “The Nogo receptor ligand LGI1 regulates synapse number and synaptic activity in hippocampal and cortical neurons” Thomas and colleagues describe a role for the NgR1 secreted competitive agonist LGl1 in regulating synaptic density during hippocampal development.

The main findings described in this manuscript are: 1) gain-and loss-of-function of LGl1 result respectively in increased and decrease synapse number in primary hippocampal neurons; 2) NgR1 derived primary hippocampal neurons show an increased synaptic density; 3) the expression of synaptic proteins in neurites is respectively reduced and increased in LGl1 and NgR1 knockout cultures; 4) the levels of active RhoA are increased in LGl1 knockout primary neurons; 5) RhoA dependent contraction in cell size is blocked by LGl1 application; 6) while spine density is not decreased in LGl1 knockout mice in vivo, the proportion of mushroom spines is reduced and spines are shorter than controls. 7) In vivo mEPSC frequency and the amplitude, although to a lower extent are reduced. Also while presynaptic transmission is normal in LGl1 knockout mice, activity evokes epileptiform-like discharges in CA1.

The novelty of this work lies in identifying a specific signaling of LGl1 via NgR1/Troy in modulating synapse number. However, this conclusion needs to be strengthened by excluding an indirect effect via ADAM22. The role of ADAM22 in synapse maturation was in fact already shown (Lovero et al., 2015).

While I find the topic interesting and the study mostly thoroughly performed, I have a couple of issues that I think should be addressed before the manuscript can be considered for publication.

Major points:

1) The authors use a gain-of-function approach for LGl1, observe an increase in synaptic density and conclude that LGl1 “potently promotes synapse formation in hippocampal neurons in vitro”. While I think that this is a possible interpretation of the results other possibilities should be considered. As described in the methods, the cultures were let develop for 18 days to an age in vitro at which cultures are considered to be essentially mature and then treated for 6 day with LGl1. I was wondering how the authors can be so sure that the increase synaptic density observed is indeed due to increase synapse formation and not rather increase stabilization of existing synapses or also reduced elimination. This question could be addressed for instance by performing time lapse imaging of primary hippocampal neurons expressing GFP tagged PSD95 or at least by analyzing the effects of LGl1 application at different times during development. Certainly this question should be discussed.

2) The authors conclude that LGl1 modulates synapse formation by directly regulating the NgR1/Troy mediated RhoA activations. However, LGl1 has been shown to bind to ADAM22 and thus modulate synapse maturation and function (Lovero et al., 2015), and the authors themselves have shown that NgR1 and ADAM22 physically associate to form a receptor complex in which NgR1 facilitates LGI1 binding to ADAM22 (Thomas et al., 2010). I was wondering how can the authors completely exclude ADAM22 from the signaling and not consider the possibility of an indirect effect via ADAM22. The conclusion would be strengthened by showing that COS7 cells do not express ADAM 22 and possibly by showing that downregulating ADAM22 in hippocampal neurons does not prevent the effect of a LGl1 application on synaptic density.

3) The spine density values for CA1 pyramidal neurons of LGl1 +/+ mice are extremely low and do not really correspond to those commonly seen in the literature. This is possibly due to the fact that the dendritic branches selected, as visible in the pictures are relatively thick leading to underestimating spine number. More accurate spine density measurements should be done by choosing thinner 2nd, 3rd order dendritic branches

Minor points:

1) In Figure 4 blots are shown both for PSD95 and Synaptophasin however, only PSD95 is quantified. Quantification should be provided for both proteins.

2) In the legend of figure 1, 2 and 3 it is written that the images show a DIV18 or DIV15 cultured hippocampal neurons. If this are neurons that underwent t6 days treatment at the end of the 18days of cultivation the age should be different? Or the experimental plan should be better explained in the methods.

3) While the graphs in figure 1, 2 and 3 show colocalized particles normalized to 1, in the results the difference is given in %.This is confusing, either the normalized values should be given in the text of legend or the graphs should be changed.

REVIEWER 2

This work demonstrates that LGI1 gain and loss-of-function modulates excitatory synapse assembly, as measured by immunocytochemistry and western blotting of markers of excitatory synapses, electrophysiological assessment of post-synaptic currents (mEPSCs) and quantification of a structural substrate of excitatory synapses, dendritic spines. Further, investigators show that paired stimulation of hippocampal neurons in LGI-1 knockouts results in epileptic-like discharges, suggesting features of hyperexcitability are already resident in these animals, consistent with seizures that will develop in these animals. Finally, investigators show RhoA activity is upregulated in neuron cultures from LGI-1 knockouts, consistent with LGI1 inhibiting NgR1 during synapse development. Work in heterologous cells show that LGI1 overexpression reverses NgR1 signaling mediated cell collapse, demonstrating LGI1 inhibits NgR1-RhoA signaling. While this work falls short in directly demonstrating LGI1 blocks NgR-mediated inhibition of synapse assembly, it advances our understanding of LGI1 function during synapse development, and provides correlative evidence suggesting LGI1 might act by targeting NgR signaling on dendrites.

We feel the authors have largely addressed our previous reviews, outside of providing direct evidence implicating LGI1 in NgR1-dependent synaptic biology. In light of this omission, we would suggest the authors change the final sentence of their abstract from “Our data indicate that LGI1 enhances synapse formation in cortical and hippocampal neurons by reducing NgR1 signaling” to, for example, “Our data suggests...” since RhoA is known to mediate the signaling of numerous synaptic receptors and a global quantification of RhoA activity such as the investigators carried out does not necessarily reflect what is happening along dendrites. Finally, we still feel the images in Figure 8A are not representative of the specific mushroom spine loss quantified in LGI1 knockouts documented in this figure. The current figure depicts profound spine loss of every spine type. Lastly, there is a typo on the bottom of page 4 “NgR1 and its coreceptor TROY are expressed also expressed in the dendrites of mouse hippocampal neurons (Wills et al., 2012).”
